# Management of Mild Postpartum Anemia: Is Iron Administration Effective?

**DOI:** 10.7759/cureus.65276

**Published:** 2024-07-24

**Authors:** Akihito Morita, Daisuke Higeta, Yoshikazu Kitahara, Maki Inoue, Akira Iwase

**Affiliations:** 1 Obstetrics and Gynecology, Gunma University Hospital, Maebashi, JPN

**Keywords:** postpartum, iron therapy, hemoglobin, cesarean section, anemia

## Abstract

Background

This study aimed to investigate the efficacy of iron therapy in the treatment of mild postpartum anemia.

Methods

We conducted a case-control study involving women who underwent cesarean section at our hospital between 2015 and 2020. Following propensity score matching, participants were categorized into two groups based on whether or not they received iron therapy. These patients were evaluated for mean hemoglobin (Hb) levels on the seventh postoperative day (POD 7), the percentage of subjects achieving Hb greater than 10 g/dL on POD 7, and the incidence of adverse events. The efficacy of iron administration was evaluated using a superiority test, and receiver operating characteristic analyses were employed to generate area under the receiver operating characteristic curves (AUROC).

Results

The mean Hb level on POD 7 was 10.12 g/dL in the iron group and 9.89 g/dL in the iron-free group (P = 0.206). The superiority test revealed that the percentage of subjects achieving Hb levels greater than 10 g/dL on POD 7 was 56.1% in the iron group and 48.8% in the iron-free group (P = 0.880), indicating that the iron group did not demonstrate superiority over the iron-free group. The incidence of adverse events was significantly higher in the iron group (P = 0.027). The highest AUROC was observed with preoperative mean corpuscular Hb, measuring 0.632 (95% CI: 0.509-0.755), with a cutoff point of 28.5 pg.

Conclusion

Consideration should be given to the uniform administration of iron for the management of mild postpartum anemia.

## Introduction

Pregnant women are frequently diagnosed with physiological anemia due to the notable increase in circulating plasma volume [1]. This increase, ranging from 30% to 40% during pregnancy, serves the dual purpose of maintaining proper fetal-placental circulation and preparing for potential postpartum hemorrhage (PPH) [2]. Given that PPH is the primary contributor to anemia during the postpartum period, instances of postpartum anemia often result from unexpected hemorrhage during delivery. Several studies indicate that postpartum anemia affects 8-50% of cases [3]. The WHO classification for pregnancy defines anemia as a hemoglobin (Hb) level of less than 11 g/dL [4]. However, the WHO classification applies during pregnancy, and there is no clear definition of postpartum anemia. Of clinical significance is when the Hb level falls below 10 g/dL [5]. Hb levels in Japanese pregnant women are lower than the WHO classification, and the definition of postpartum anemia in Japanese pregnant women may be even lower [6].

Patients undergoing cesarean section (CS) are at an increased likelihood of requiring treatment for anemia because CS is associated with higher blood loss than vaginal delivery. In clinical practice, blood tests are generally performed on the first postoperative day after CS to screen for anemia. If a patient is diagnosed with anemia and does not require a blood transfusion, treatment options such as iron therapy are recommended. However, the diagnosis of anemia and the indication for iron therapy vary based on the criteria of individual institutions. While several studies have explored the use of iron in treating postpartum anemia, the efficacy of iron therapy for mild postpartum anemia remains controversial [7-9]. Furthermore, the evaluation of anemia and the determination to initiate iron therapy included parameters such as Hb levels, serum ferritin levels, and erythrocyte indices (i.e., mean corpuscular volume (MCV), mean corpuscular Hb (MCH), and MCH concentration (MCHC)). However, there is currently no consensus on which indicators should be prioritized in assessing and managing postpartum anemia.

Mild postpartum anemia is characterized by a few subjective symptoms, and patients typically experience a swift recovery. A previous study reported that adverse events occur in 10-20% of patients receiving oral iron [9]. Nevertheless, in clinical practice, there is a common tendency to uniformly administer iron therapy, even in cases of mild postpartum anemia. Given the potential adverse effects associated with iron therapy, it is contentious whether a uniform treatment approach solely based on Hb levels is appropriate for these patients. This study aimed to investigate the effectiveness of iron therapy, specifically in cases of mild postpartum anemia following CS.

## Materials and methods

Study design

This single-center, retrospective observational study was conducted at Gunma University Hospital in Maebashi, Japan, and received approval from the Institutional Review Board of Gunma University Hospital (approval number HS2019-134). The medical records of patients who underwent CS between April 2015 and March 2020 at Gunma University Hospital were reviewed. The study retrospectively explored the efficacy of iron-based therapy in terms of mean Hb levels on the seventh postoperative day (POD 7), the percentage of subjects achieving Hb levels greater than 10 g/dL on POD 7, and the incidence of adverse events in eligible cases.

Participants

All patients who underwent cesarean delivery during the study period were included in the analysis. The exclusion criteria comprised (1) patients requiring blood transfusion or autologous blood transfusion; (2) patients discharged within POD 5; and (3) patients with Hb levels either <9.0 g/dL or ≥10.0 g/dL on POD 1. The cutoff value in this study was set to 9.0 g/dL on POD 1 based on the observation that most patients with Hb levels <9.0 g/dL on POD 1 received iron treatment at Gunma University Hospital. Conversely, cases with Hb levels >10 g/dL on POD 1 were not treated. Furthermore, the decision to continue further iron therapy is based on the Hb levels on POD 7. The definition of postpartum anemia in Japanese women is unclear, and in general, Japanese pregnant women have low Hb levels [6]. Therefore, Hb levels of 10 g/dL on POD 1 were established as the cutoff point for inclusion, and mild postpartum anemia was defined as a postpartum Hb level between 9 g/dL and 10 g/dL in this study. Iron therapy, 100 mg of sodium ferrous citrate, was initiated within 24 hours after delivery and administered daily throughout the hospitalization period.

Statistical analysis

Propensity score matching (PSM) was performed. A three-to-one matching without replacement was completed using the nearest neighbor match on the logit of the propensity score. The caliper width was set to 0.05 times the SD of the logit of the propensity score. The propensity score was determined using a logistic regression model with iron administration as the dependent variable, while potential confounders, including preoperative iron administration, blood loss during CS, and Hb levels on POD 1, were the independent variables. Data were expressed as mean. Comparisons between the iron and iron-free groups for each parameter were performed using the unpaired t-test, chi-squared test, or Fisher’s exact test. The efficacy of iron therapy was evaluated using a superiority test, with a superiority margin of 20%. The test evaluated the superiority of the iron group over the iron-free group. A one-sided 90% CI was provided, and statistical significance was set at P < 0.05. The efficacy of postpartum iron therapy was evaluated using the area under the receiver operating characteristic curve (AUROC) for intraoperative blood loss, MCV, MCH, and MCHC before CS and on POD 1, as part of a sensitivity analysis. The optimal cutoff point was defined as the maximum sum of sensitivity and specificity using the Youden index approach. Statistical analyses were performed using IBM SPSS Statistics for Windows, Version 29.0 (Released 2022; IBM Corp., Armonk, NY, USA) and SAS version 9.4 (SAS Institute Inc., Cary, NC, USA). All tests were two-tailed, and statistical significance was set at a P-value < 0.05.

## Results

A total of 805 women underwent CS during the study period. Of these patients, 95 required transfusions or autologous blood transfusions. Additionally, 24 patients were discharged within POD 5, and 529 patients with Hb levels either <9.0 g/dL or ≥10.0 g/dL on POD 1 were excluded from the study. Ultimately, 157 patients met the eligibility criteria. The eligible patients were then categorized into two groups: the iron group (n = 107), consisting of patients who received iron therapy, and the iron-free group, comprising patients who did not receive iron therapy (n = 50).

The baseline characteristics are presented in Table 1. Most baseline characteristics did not exhibit statistically significant differences between the groups. However, the mean Hb levels on POD 1 were 9.42 g/dL in the iron group and 9.62 g/dL in the iron-free group, revealing a significant difference (P < 0.001). Moreover, the percentages of preoperative iron administration were 16.8% in the iron group and 4.0% in the iron-free group, showing a significant difference (P = 0.025). Therefore, PSM analysis was conducted, resulting in no statistically significant differences in baseline characteristics, excluding the gestational age at CS. Ultimately, the analysis included 41 patients in both the iron and iron-free groups.

**Table 1:** Characteristics of each group Values are presented as mean (SD). CS, cesarean section; Hb, hemoglobin levels; POD1, first post-operative day; PSM, propensity score matching

Variables	Observed data			PSM data		
	Iron (n = 107)	Iron-free (n = 50)	P-value	Iron (n = 41)	Iron-free (n = 41)	P-value
Maternal age (years)	32.9 (5.0)	33.6 (5.3)	0.416	33.1 (4.5)	33.9 (5.5)	0.487
Multiparity, n (%)	61 (57.0)	22 (44.0)	0.128	22 (53.7)	16 (39.0)	0.184
Parity (times)	0.8 (0.9)	0.6 (0.8)	0.204	0.9 (1.1)	0.5 (0.7)	0.054
Interpregnancy interval (months)	30.2 (39.7)	19.9 (29.8)	0.105	26.0 (37.0)	16.3 (26.3)	0.176
Hb levels on POD1 (g/dL)	9.42 (0.27)	9.62 (0.23)	<0.001	9.58 (0.25)	9.60 (0.25)	0.755
Gestational age at CS (weeks)	37.2 (3.4)	36.8 (3.6)	0.495	38.1 (1.7)	36.9 (3.5)	0.048
Emergency CS, n (%)	57 (53.3)	28 (56.0)	0.749	19 (46.3)	25 (61.0)	0.184
Preoperative iron administration, n (%)	18 (16.8)	2 (4.0)	0.025	1 (2.4)	2 (4.9)	0.5
Intraoperative blood loss (g)	1,117 (592)	1,173 (527)	0.572	1,194 (719)	1,125 (488)	0.614

**Figure 1:**
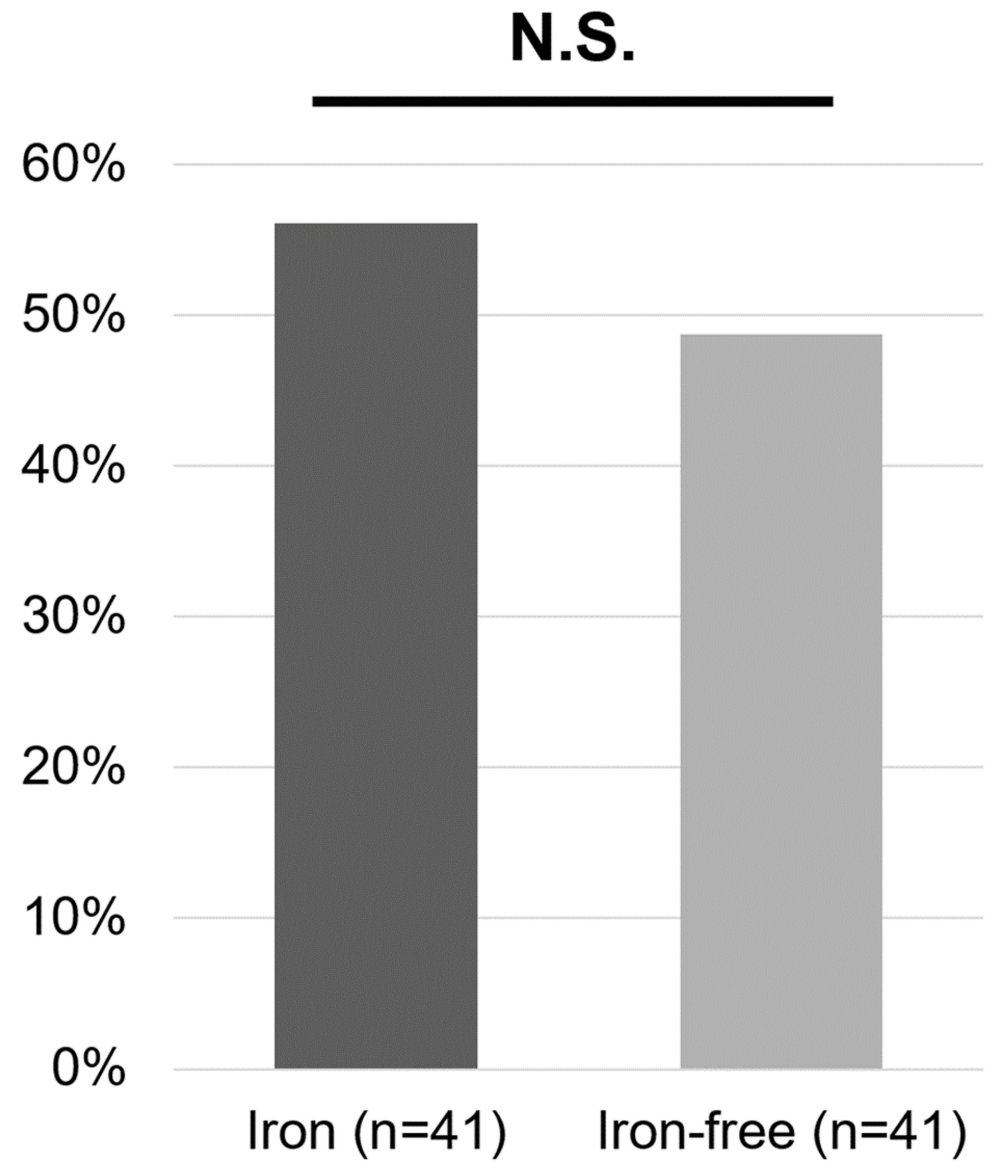
Percentage of patients who achieved Hb levels greater than 10 g/dL (treatment target value) on POD 7 Hb, hemoglobin; POD 7, seventh postoperative day

Table 2 shows the mean Hb levels on POD 7, which were 10.12 g/dL in the iron group and 9.89 g/dL in the iron-free group (P = 0.206). A superiority test was conducted on the achievement rate of the treatment target (Figure 1). The percentage of patients achieving the treatment target was 56.1% in the iron group and 48.8% in the iron-free group, indicating that the superiority of the iron group was not established (difference = 0.073; 90% CI: -0.105 to 0.251; P = 0.880 for superiority). Additionally, adverse events were observed in 12.2% of patients in the iron group, while no adverse events occurred in the iron-free group (Table 2). Among the adverse events, 80.0% were associated with hepatic dysfunction, and 20.0% involved nausea.

**Table 2:** Postpartum Hb levels and adverse event Values are presented as mean (SD). Hb, hemoglobin; POD7, seventh post-operative day

Parameter	Iron (n = 41)	Iron-free (n = 41)	P-value
Mean Hb levels on POD7 (g/dL)	10.12 (0.72)	9.89 (0.88)	0.206
Adverse events, n (%)	5 (12.2)	0 (0.0)	0.027

**Figure 2:**
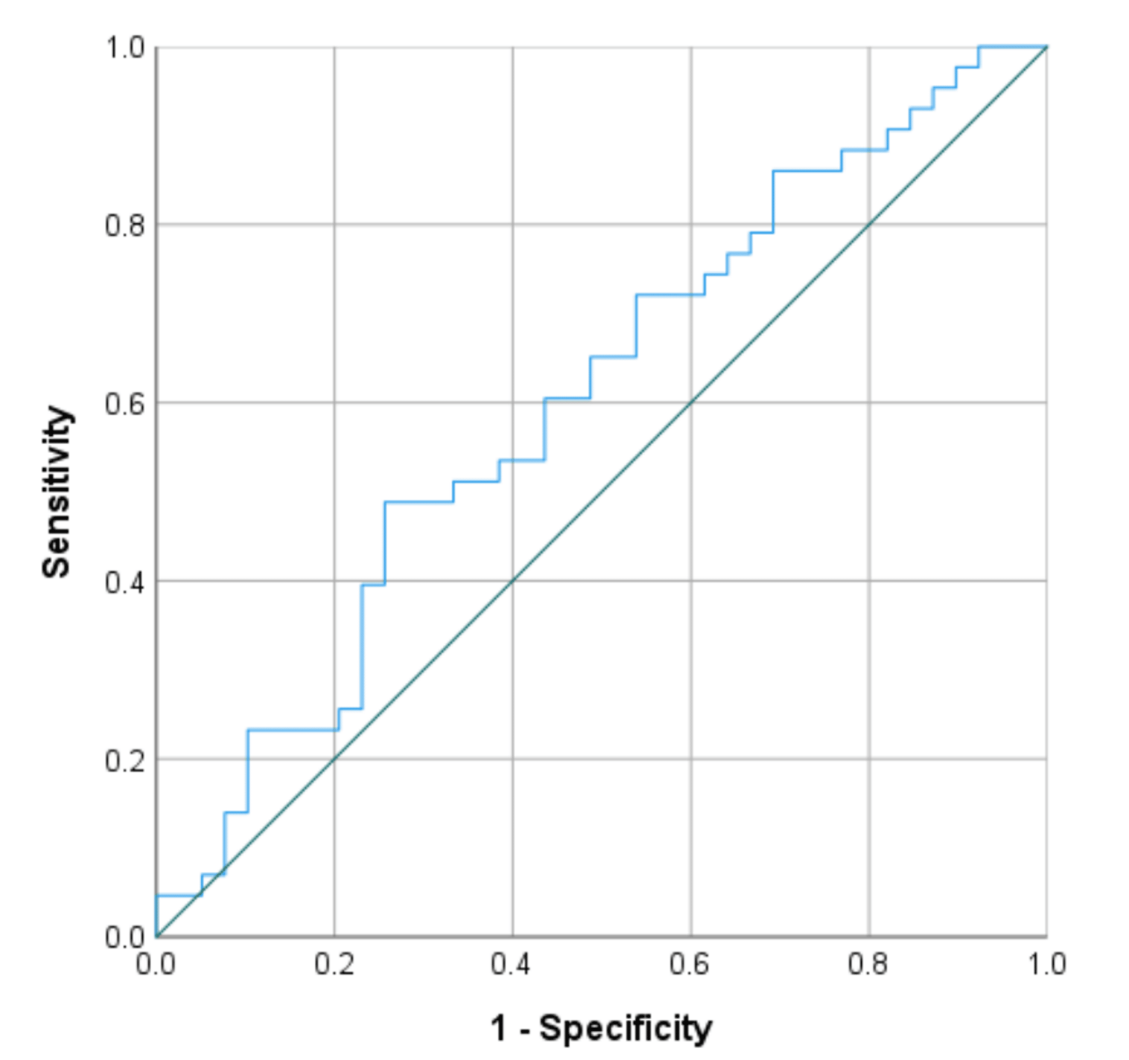
ROC curve analysis for iron administration in intraoperative blood loss ROC, receiver operating characteristic

**Figure 3:**
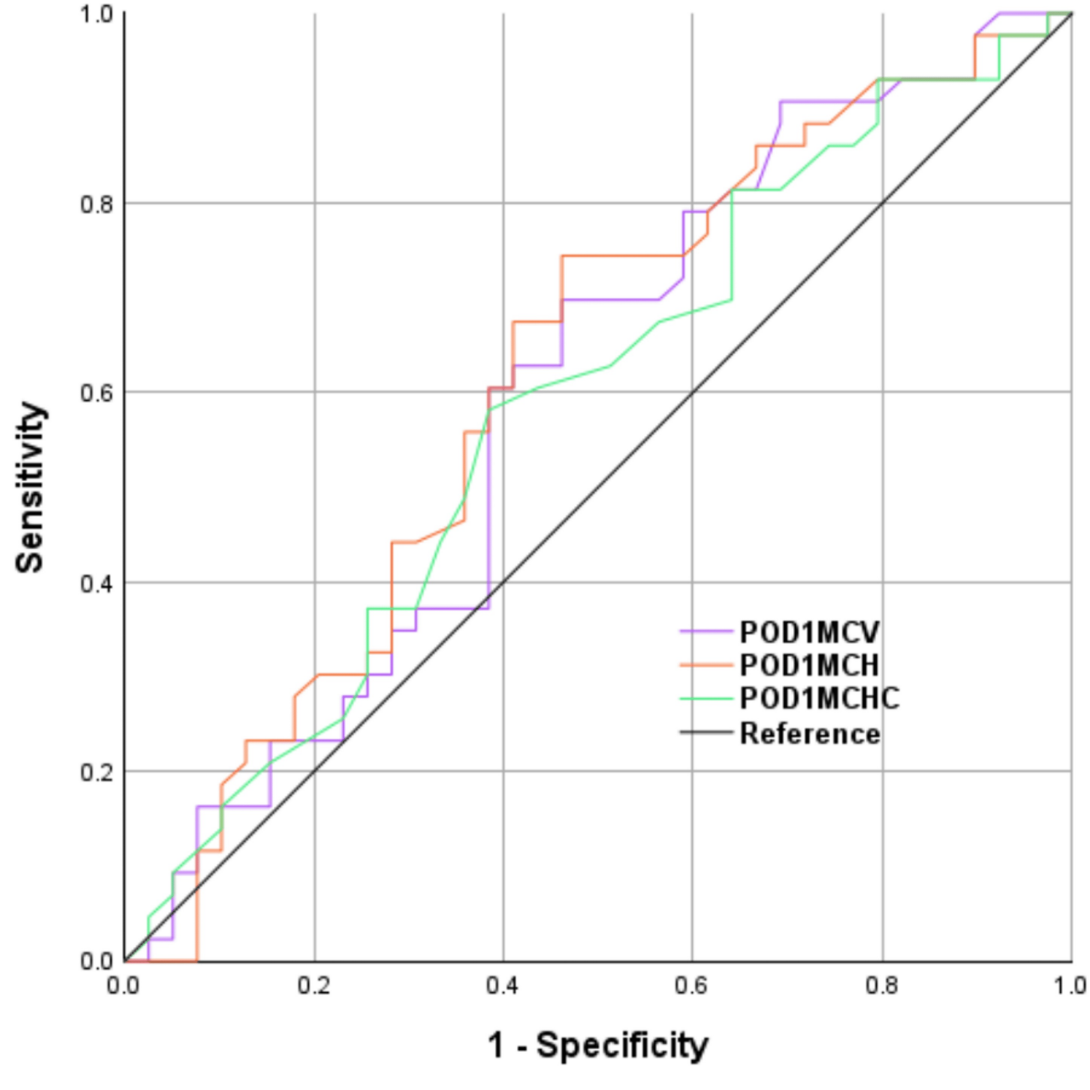
ROC curve analysis for iron administration in each of the preoperative erythrocyte indices POD1, first post-operative day; MCV, mean corpuscular volume; MCH, mean corpuscular hemoglobin; MCHC, mean corpuscular hemoglobin concentration

The ROC curve for intraoperative blood loss is illustrated in Figure 2, displaying an AUROC of 0.603 (95% CI: 0.480-0.727) and a cutoff point of 1,184 mL. The ROC curves for preoperative parameters (MCH, MCHC, and MCV) before CS are depicted in Figure 3. The AUROC and cutoff points for preoperative MCH were 0.632 (95% CI, 0.509-0.755) and 28.5 pg, for MCHC were 0.599 (95% CI, 0.475-0.724) and 33.2%, and for MCV were 0.613 (95% CI, 0.489-0.737) and 87.0 fL. The AUROC and cutoff points for MCH on POD 1 were 0.616 (95% CI, 0.492-0.741) and 28.4 pg, for MCHC on POD 1 were 0.586 (95% CI, 0.460-0.711) and 32.4%, and for MCV on POD 1 were 0.597 (95% CI, 0.471-0.723) and 85.8 fL.

## Discussion

In this study, we did not observe a superior efficacy of iron therapy over the iron-free approach in the management of mild postpartum anemia with Hb levels at 9.0 g/dL. The mean Hb levels and the percentage of treatment targets achieved on POD 7 were comparable between the two groups. Adverse events associated with iron therapy were observed in 12.2% of patients. Furthermore, our findings suggest that the potential use of iron therapy may be more pronounced when the preoperative MCH is lower than 28.5 pg.

Iron deficiency anemia during pregnancy is associated with various adverse effects on both mothers and infants. In mothers, it has been reported to increase the risk of CS and necessitate blood transfusions and preterm delivery [10-12]. For infants, the consequences include an increased risk of low birth weight, macrosomia, preterm delivery, perinatal mortality, and neurodevelopmental disorders [11]. A recent Japanese report highlighted an association between anemia during pregnancy, preterm delivery, and small-for-gestational-age infants [6]. However, when anemia, particularly mild anemia, is initially diagnosed in the postpartum period, a dispute arises regarding whether therapeutic intervention can improve outcomes for both the mother and child.

Previous studies have compared different routes of iron therapy or assessed iron therapy in conjunction with erythropoietin. Few reports have specifically addressed the effectiveness of iron therapy alone. However, given the postpartum anemia treatment limitations, particularly in Japan, clinicians may themselves opt for iron therapy as a primary prevention. Adverse events associated with oral iron agents occur in 10-20% of patients, with gastrointestinal symptoms being the most common [9]. In comparison, intravenous administration offers a higher and faster rate of increase in Hb levels and a higher increase in serum ferritin levels [13]. Therefore, intravenous iron administration may be a viable alternative to iron therapy. In this study, gastrointestinal symptoms were infrequent, while hepatic dysfunction occurred more frequently. We attribute these differences in adverse events to the retrospective analysis, which made it difficult to identify potential symptoms in patients. Considering the frequency of adverse events, iron therapy should be reconsidered for mild postpartum anemia with minimal subjective symptoms.

We were unable to establish the superiority of the iron group over the iron-free group in the context of mild postpartum anemia. However, anemia during pregnancy and the postpartum period has associations with postpartum depression, and iron intake serves as a critical nutritional requirement due to relative iron deficiency during this period [14,15]. Although an inverse association between postpartum Hb levels and postpartum depressive symptoms has been reported, the difference was not significant in cases of mild anemia [16]. The question arises: Is iron therapy truly unnecessary for all patients with mild postpartum anemia? While accurate diagnosis of iron deficiency typically requires blood tests such as serum ferritin levels and iron-binding capacity, the practicality of performing these tests for all patients is challenging, as in this study. Iron deficiency anemia, characterized by low MCV and MCH, is the most prevalent form of microcytic hypochromic anemia. Although MCV is commonly used for diagnosing microcytic anemia, our study identified MCH as a statistically significant marker. The strengths of this study include its single-center design and the practical application of easily measurable parameters (MCV and MCH) in routine clinical practice. In recent years, new formulations of iron agents with reported fewer adverse events have emerged, but their widespread adoption has been hindered by high costs [17]. By identifying a subgroup of patients who may benefit from iron therapy without additional examinations, our study offers a practical approach to intervention as an extension of daily clinical practice.

This study has some limitations. First, the evaluation of iron therapy efficacy was based on Hb levels on POD 7. This timeframe might be too early to accurately assess efficacy. Reticulocyte count typically increases within a few days, and Hb levels start to increase approximately one week after the initiation of oral iron administration. Second, although previous reports suggest that iron supplementation during pregnancy reduces postpartum anemia, a notable imbalance was observed in our study, with significantly more cases in the iron group having received preoperative iron therapy [18]. Thus, PSM analysis was used to mitigate the impact of this factor. However, we cannot entirely rule out the potential bias introduced by preoperative iron administration in influencing the decision to initiate postoperative iron therapy. Third, this study did not account for complications such as preeclampsia and the patient's income or occupation and may not have adequately examined confounding factors that affect anemia. Finally, the timing of blood sampling for determining the decision to administer iron was based on morning POD 1 Hb levels, and the results might have been influenced by differences in crystalloid solution infusion volume due to the specific timing of CS.

## Conclusions

In our present study, we were unable to demonstrate the superiority of the iron group over the iron-free group in the context of mild postpartum anemia. Additionally, we observed several adverse events associated with iron therapy. Furthermore, our findings suggest that iron therapy may be more beneficial when the preoperative MCH is lower than 28.5 pg. This emphasizes the importance of considering the necessity of iron therapy not only in relation to postoperative Hb levels but also in light of other laboratory findings, including MCH. Our study suggests that the practice of uniformly administering iron therapy for mild postpartum anemia should be reconsidered.
